# Disorder Scattering Induced Large Room Temperature Nonlinear Anomalous Hall Effect in a Semiconductor CdGeAs_2_

**DOI:** 10.1002/adma.202514217

**Published:** 2025-11-23

**Authors:** Seng Huat Lee, Takumi Iwaya, Kosuke Nakayama, Ting Yong Lim, Lujin Min, Jingyang He, Yu Wang, Venkatraman Gopalan, Zhijian Xie, Xing‐Chen Pan, Yong P. Chen, Tay‐Rong Chang, Hsin Lin, Liang Fu, Kouji Segawa, Takafumi Sato, Zhiqiang Mao

**Affiliations:** ^1^ 2D Crystal Consortium Materials Research Institute The Pennsylvania State University University Park Pennsylvania 16802 USA; ^2^ Department of Physics The Pennsylvania State University University Park PA 16802 USA; ^3^ Department of Physics Graduate School of Science Tohoku University Sendai 980‐8578 Japan; ^4^ Department of Electrical and Department of Physics National Cheng Kung University Tainan 70101 Taiwan; ^5^ Department of Materials Science and Engineering The Pennsylvania State University University Park PA 16802 USA; ^6^ Department of Electrical and Computer Engineering North Carolina Agriculture and Technical State University Greensboro NC 27411 USA; ^7^ Advanced Institute for Materials Research (WPI‐AIMR) Tohoku University Sendai 980‐8577 Japan; ^8^ Department of Physics and Astronomy Elmore Family School of Electrical and Computer Engineering Birck Nanotechnology Center Purdue Quantum Science and Engineering Institute Purdue University West Lafayette IN 47906 USA; ^9^ Institute for Physics and Astronomy and Villum Center for Hybrid Quantum Materials and Devices Aarhus University Aarhus‐C 8000 Denmark; ^10^ Center for Quantum Frontiers of Research and Technology (QFort) Tainan 70101 Taiwan; ^11^ Physics Division National Center for Theoretical Sciences Taipei 10617 Taiwan; ^12^ Institute of Physics Academia Sinica Taipei 115201 Taiwan; ^13^ Department of Physics Massachusetts Institute of Technology Cambridge MA 02139 USA; ^14^ Department of Physics Kyoto Sangyo University Kyoto 603‐8555 Japan

**Keywords:** CdGeAs_2_, frequency mixing, nonlinear hall effect, semiconductor

## Abstract

The nonlinear Hall effect (NLHE) with time‐reversal symmetry has emerged as a transformative phenomenon within the Hall effect family, attracting significant interest due to its profound implications for both fundamental physics and technological applications. While prior studies have predominantly focused on NLHE in 2D materials, advancements in practical applications have been constrained by low operating temperatures and limited responsivity, typically below 10^−4^ m/V. Achieving significant responsivity at room temperature (RT) in 3D systems has proven challenging, particularly for scattering‐induced NLHE. Here, the discovery of disorder scattering‐induced NLHE in chalcopyrite‐type CdGeAs_2_ bulk single crystals is reported, demonstrating a remarkable responsivity of up to 10^−3^ m/V at RT. The studies reveal that NLHE not only facilitates ac‐driven second harmonic and rectification Hall responses but also induces an exceptionally large anomalous Hall angle. Through band structure measurements by ARPES, DFT calculations, as well as symmetry and nonlinear Hall conductivity scaling analyses, disorder scattering is identified as the dominant mechanism for the NLHE in CdGeAs_2_. Leveraging the observed strong responsivity of NLHE at RT, its broadband electronic frequency mixing capability in the MHz range is further demonstrated. This work sets the foundation for integrating scattering‐induced NLHE in 3D materials into very high‐frequency mixing technologies.

## Graphical abstract

Bulk CdGeAs_2_, a well‐known nonlinear optical semiconductor, exhibits an exceptionally large room‐temperature nonlinear Hall effect (NLHE), achieving a record‐high nonlinear Hall responsivity of 2.2 × 10^−3^ m/V. This pronounced NLHE enables broadband frequency mixing up to the megahertz regime, establishing CdGeAs_2_ as a promising platform for next‐generation nonlinear electronic and optoelectronic technologies.

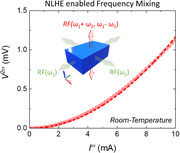

## 1 Introduction

As a new member of the Hall effect family, the nonlinear Hall effect (NLHE) has recently attracted substantial attention due to its occurrence in time symmetry invariant systems.^[^ ^1^, ^2^, ^3^, ^4^, ^5^, ^6^, ^7^, ^8^, ^9^, ^10^ ^]^ In the linear response regime, the Hall conductivity requires breaking time‐reversal symmetry through magnetization or an external magnetic field. In contrast, under a nonlinear response regime, Hall response can occur in materials that preserve time‐reversal symmetry, provided inversion symmetry is broken. This intriguing phenomenon, known as NLHE, allows for the conversion of longitudinal alternating current (AC) at a specific frequency (*ω*) into a second harmonic (2*ω*) AC Hall voltage and a rectified direct current (DC) Hall voltage, all without the need for an external magnetic field. This unique characteristic significantly broadens the scope of Hall effect studies, opening new avenues for exploration in both fundamental physics and technological applications.

Intrinsic NLHE was initially proposed to arise from the Berry curvature dipole (BCD), where inversion symmetry breaking causes the segregation of positive and negative Berry curvatures across different momentum regions, thereby generating a dipole moment.^[^ ^1^ ^]^ This mechanism makes NLHE a particularly compelling phenomenon in the study of topological materials. Experimental observations of BCD‐induced NLHE have been reported in a range of 2D topological materials and related systems, including bilayer WTe_2_,^[^ ^3^ ^]^ few‐layer WTe_2_,^[^ ^4^ ^]^ corrugated bilayer graphene,^[^ ^5^ ^]^ monolayer WSe_2_,^[^ ^11^ ^]^ twisted bilayer WSe_2_,^[^ ^12^ ^]^ NbIrTe_4_,^[^ ^13^ ^]^ and Pb_1‐_ *_x_* Sn*_x_* Te.^[^ ^14^ ^]^ It has also been observed in several 3D materials, such as Cd_3_As_2_,^[^ ^15^ ^]^ Ce_3_Bi_4_Pd_3_,^[^ ^16^ ^]^ Pb_1‐_ *_x_* Sn*_x_* Te,^[^ ^17^ ^]^ BaMnSb_2_,^[^ ^8^ ^]^ and GeTe.^[^ ^18^ ^]^ A recent breakthrough in this field is the discovery of quantum metric‐induced NLHE in the intrinsic antiferromagnetic topological insulator MnBi_2_Te_4_^[^ ^9^, ^10^, ^19^ ^]^ and Mn_3_Sn/Pt heterostructure.^[^ ^20^ ^]^ The quantum metric refers to the real part of the quantum geometry tensor, which describes the variation of a wavepacket's geometric shape. Quantum metric‐induced NLHE is independent of scattering time (*τ*), which is believed to be advantageous for terahertz (THz) detection, as the BCD‐induced NLHE depends on *τ* and imposes a frequency limit at 1/*τ*. In addition to the intrinsic NLHE effect induced by band topology, extrinsic NLHE due to various mechanisms has also been found in a range of materials such as graphene/BN moiré superlattices,^[^ ^21^ ^]^ twisted bilayer graphene,^[^ ^22^ ^]^ *T*_d_‐MoTe_2_,^[^ ^23^ ^]^ TaIrTe_4_,^[^ ^6^ ^]^ Bi_2_Se_3_,^[^ ^24^ ^]^ Bi_2_Te_3_,^[^ ^25^ ^]^ 1*T*‐CoTe_2_,^[^ ^26^ ^]^ PbTaSe_2_,^[^ ^27^ ^]^ MBE‐grown MnBi_2_Te_4_,^[^ ^28^ ^]^ MnBi_4_Te_7_,^[^ ^29^ ^]^ Bi polycrystalline thin films,^[^ ^30^ ^]^ Te‐thin films,^[^ ^31^ ^]^ Pt nano texture^[^ ^32^ ^]^ and etc.^[^ ^33^ ^]^ The NLHE in these materials originates from either skew scattering and/or side‐jump in the thin film or surface of the bulk material, directly from the bulk, or from geometric asymmetric scattering of textured nanoparticles. Regardless of the origin, all extrinsic origin‐induced NLHE is characterized by dependence on *τ* ^2^. The nonlinear Hall conductivity for intrinsic and extrinsic NLHE follows different scaling behaviors with *τ*.^[^ ^34^, ^35^ ^]^

In addition to its scientific relevance in exploring quantum geometry, the NLHE holds significant promise for practical applications in frequency‐doubling and rectifying devices. Functioning as a rectifier, the device converts an oscillating longitudinal input into a unidirectional (DC) transverse voltage, thereby generating DC power. Of particular interest are NLHE‐based wireless rectifiers, which are highly attractive for energy harvesting and wireless charging applications, benefiting from their broadband response and ability to function in the absence of an external magnetic field. However, the predominance of 2D materials, which typically operate at low temperatures, has hindered their practical implementation. Achieving high NLHE responsivity (defined as $R=E_{⊥}^{2\omega }/{\left(E_{\left|\right|}^{\omega }\right)}^{2}$ where $E_{\;⊥}^{2\omega }$ and $E_{\;||}^{\omega }$ represents the values of transverse and longitudinal electric fields, respectively) at room temperature without complex device fabrication is critical for technological advancement. Several 2D and 3D materials have been found to exhibit room‐temperature NLHE (see **Table** **1**). Among them, those 2D materials exhibit weak NLHE response, with *R* ranging from 10^−10^ to 10^−8^ m/V, irrespective of their underlying mechanism, whereas two 3D materials, i.e., GeTe and BaMnSb_2_, display strong NLHE associated with the BCD, with *R* being at the order of 10^−4^ m/V at room temperature. The geometrically asymmetric scattering‐induced extrinsic NLHE in Pt textured nanoparticles exhibits an even stronger response, with *R* ∼ 10^−3^ m/V. While disorder scattering has been shown to give rise to even higher NLHE responsivity, reaching up to 10^−1^ m/V in *T*_d_‐MoTe_2_,^[^ ^23^ ^]^ it is present only at low temperatures. The potential to achieve disorder scattering‐induced NLHE in a 3D material with substantial responsivity at room temperature remains an open challenge. In this study, we report an exceptionally strong disorder scattering‐induced NLHE with a record responsivity (*R* = 2.2 × 10^−3^ m/V) at room temperature, which is observed in a chalcopyrite bulk crystal CdGeAs_2_.

**Table 1:** Comparison of NLHE's origin, responsivity, and the second‐order Hall voltage response for materials exhibiting the room‐temperature NLHE.

Compound	Dimensionality	Origin	2^nd^ Order voltage response	Responsivity [m/V]	Refs.
TaIrTe_4_	2D (20 nm)	Surface	100 µV at 600 µA	2.5 × 10^−8^	[6]
Pb_1‐_ *_x_* Sn*_x_* Te	2D	BCD	250 µV at 100 µA		[14]
MnBi_2_Te_4_	2D (10–30 nm)	Spin‐orbit scattering	225 µV at 250 µA	3.0 × 10^−10^	[28]
Bi polycrystalline thin films	2D (100 nm)	Disorder and geometry asymmetry scattering	3 µV at 60 µA	2.4 × 10^−9^ (Estimated)	[30]
Te‐thin flakes	2D (20–30 nm)	Scattering	2800 µVat 50 µA	8.0 × 10^−8^	[31]
Bi_2_Te_3_	2D (10 nm)	Scattering	250 µV at 900 µA	3.5 × 10^−9^	[25]
Mn_3_Sn/Pt	2D (30 nm)	Quantum metric	35 µV at 4500 µA		[20]
BaMnSb_2_	3D	BCD	250 µV at 100 µA	8.5 × 10^−4^	[8]
GeTe	3D	BCD	1 µV at 5 mA	2.0 × 10^−4^ (Estimated)	[18]
Pt nanoparticle	3D	Geometry asymmetry scattering	90 µV at 50 µA	1.4 × 10^−3^	[32]
CdGeAs_2_	3D	Disorder Scattering	1200 µV at 10 mA	2.2 × 10^−3^	This work

CdGeAs_2_, an $A^{\mathrm{II}}B^{\mathrm{IV}}C_{2}^{V}$‐type chalcopyrite crystallizes in the non‐centrosymmetric structure with the space group $I\bar{4}2d$.^[^ ^36^ ^]^ This material has garnered significant attention due to its exceptional nonlinear optical properties, making it highly valuable for optical devices and laser technology. CdGeAs_2_ possesses the highest nonlinear optical constant (*d*_36_ = 236 pm/V) among chalcopyrite, a large birefringence (*n*_e_ ‐ *n*_o_ = 0.09), and a wide transmission range (2.3–18 µm)^[^ ^37^, ^38^, ^39^ ^]^ together with a reasonably high thermal conductivity (4.18 W/mK).^[^ ^40^ ^]^ These characteristics make it particularly suitable for applications such as frequency doubling and mixing optical parametric oscillators.^[^ ^41^, ^42^ ^]^ Beyond its optical properties, CdGeAs_2_ has been predicted to be a topological insulator,^[^ ^43^ ^]^ although experimental verification has not been reported, potentially hosting a novel magnetoelectric effect, further enhancing its potential in advanced technological applications. However, our ARPES measurements, supported by theoretical calculations, did not reveal evidence of topological properties in CdGeAs_2_, as discussed below.

We synthesized CdGeAs_2_ single crystals using the flux growth method; its structure and composition were confirmed by X‐ray diffraction and energy dispersive spectroscopy (see Note S1, Supporting Information). Through optical second harmonic generation (SHG), we show that the as‐grown CdGeAs_2_ crystals do possess broken inversion symmetry, which leads to the NLHE. Our transport measurements reveal not only ac‐driven second harmonic and rectification Hall responses but also a large nonlinear anomalous Hall response driven by a direct current (DC). Notably, we observed that the NLHE in CdGeAs_2_ reaches its maximum amplitude near room temperature, with a responsivity *R* reaching a record value of 10^−3^ m/V. Our symmetry and scaling analyses indicate that disorder scattering plays a key role in generating the NLHE response. Finally, we demonstrate that the NLHE enables broadband electronic frequency mixing in the MHz range. This work paves the way for practical applications based on scattering‐induced NLHE in 3D materials.

## 2 Results and Discussion

### 2.1 Observation of Nonlinear Hall Effect with Large Responsivity at Room Temperature

Previously reported CdGeAs_2_ crystals have been synthesized using a low‐temperature directional crystallization technique^[^ ^44^, ^45^ ^]^ and modified vertical Bridgman method, typically resulting in a predominance of *p*‐type carriers and metallic behavior in conductivity.^[^ ^46^, ^47^ ^]^ We employed flux growth to synthesize CdGeAs_2_ crystals and demonstrated that the grown crystals retain broken inversion symmetry through optical second‐harmonic generation measurements (see Note S2, Supporting Information). Additionally, the as‐grown single crystals exhibit insulating behavior and *n*‐type carrier with a Hall carrier density of 10^16^ cm^−3^ and carrier mobilities exceeding 1000 cm^2^/Vs (see Note S3, Supporting Information).

**Figure 1:**
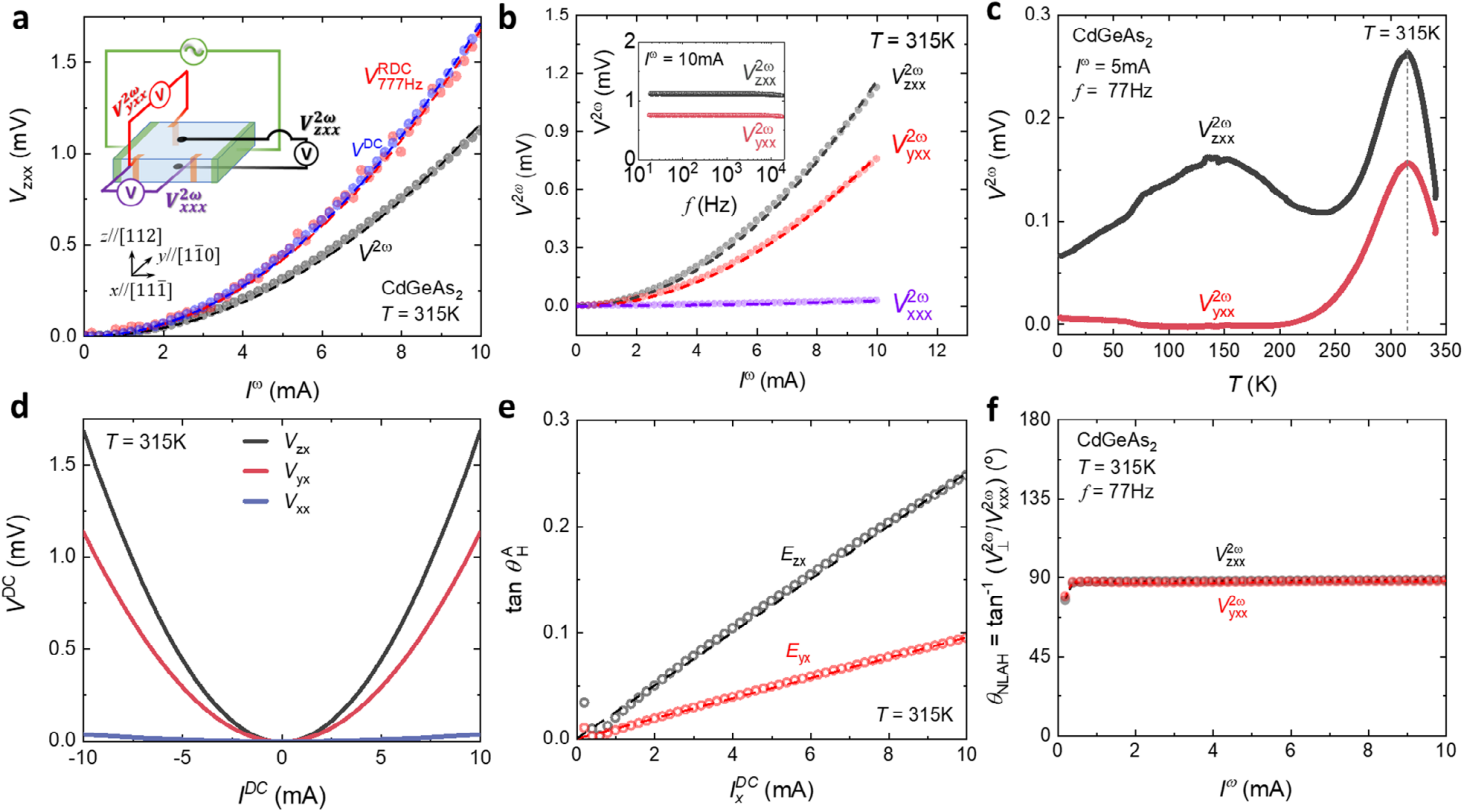
Strong room temperature NLHE of CdGeAs_2_. a) The second harmonic Hall voltage $V_{zxx}^{2\omega }$, rectified Hall voltage $V_{zxx}^{RDC}$, and DC‐driven nonlinear Hall voltage *V^DC^* at 315 K. The dots and the dashed lines are the measured data and the quadratic fit, respectively. The inset shows the experimental setup and the orientation of the crystallographic axes relative to the laboratory coordinate system. b) Nonlinear longitudinal voltage $V_{xxx}^{2\omega }$ and transverse voltage $V_{zxx}^{2\omega }$ and $V_{yxx}^{2\omega }$ as a function of input AC current *I* ^ω^ measured at 315 K, 77 Hz. The dashed lines are the quadratic fits. Inset: $V_{zxx}^{2\omega }$ and $V_{yxx}^{2\omega }$ as a function of frequency measured at *I* ^ω^ = 10 mA. c) Nonlinear transverse $V_{zxx}^{2\omega }$ and $V_{yxx}^{2\omega }$ voltage as a function of temperature measured at *I* ^ω^ = 5 mA. d) The voltages (*V* ^DC^) measured along the longitudinal and transverse directions under DC currents at 315 K. e) $\tan(\theta_{H}^{A})$ versus DC current $I_{x}^{DC}$ ($\theta_{H}^{A}$: Nonlinear anomalous Hall angle). The dashed lines in (e) are the linear fitted lines. f) Nonlinear Hall angle θ_*NLAH*_ for both transverse directions.

Next, we show that our CdGeAs_2_ crystals exhibit a strong room temperature nonlinear Hall response. We conducted nonlinear transport measurements by applying an AC current (*I^ω^*) with frequency *ω* along the $\left[11\bar{1}\right]$ direction on the (112) plane of a CdGeAs_2_ crystal (see the inset to **Figure** **1**a for the experimental setup). We measured the second‐harmonic voltage response along both the longitudinal and transverse (Hall) direction using a lock‐in amplifier technique. The second‐harmonic Hall voltage response, denoted as $V_{\;zxx}^{2\omega }$, is observed in the absence of a magnetic field and exhibits a quadratic dependence on *I^ω^* (Figure 1a). Notably, the value of $V_{\;zxx}^{2\omega }$ reached 1.2 mV at 10 mA, significantly exceeding the previously reported second‐order Hall voltage in the bulk materials exhibiting NLHE (see Table 1). Additionally, we investigated the rectified Hall voltage $V_{\;zxx}^{RDC}$ and the DC‐driven Hall voltage *V* ^DC^, both of which are proportional to (*I* ^ω^)^2^ with the expected value of $V_{\;zxx}^{RDC}=V^{DC}=\sqrt{2}V_{\;zxx}^{2\omega }$ (Figure 1a), similar to that observed in the 3D Weyl‐Kondo semimetal Ce_3_Bi_4_Pd_3_^[^ ^16^ ^]^ and Dirac material BaMnSb_2_.^[^ ^8^ ^]^

In principle, the nonlinear response is governed by the nonlinear susceptibility tensor. χ_*a*; *bc*_, where the subscripts *a*, *b*, and *c* correspond to crystallographic directions. Given that the point group of CdGeAs_2_ is $\bar{4}2m$, three independent diagonal BCD tensor components *D_aa_*, *D_bb_*, and *D_cc_* are allowed, while all non‐diagonal components vanish (see Note S4, Supporting Information). However, due to the 3D nature of the crystal, $V_{\;⊥}^{2\omega }$ and $V_{\;||}^{2\omega }$ signals measured in our experiments would arise from the combined projected components of all the allowed second‐order responses.

Given the BCD symmetrical constraints in CdGeAs_2,_ as discussed above, a nonlinear response is expected in other directions. To explore this, we measured the longitudinal second‐harmonic voltage. $V_{\;xxx}^{2\omega }$ and the transverse voltage $V_{\;yxx}^{2\omega }$ using the same device used for $V_{\;zxx}^{2\omega }$ measurements, as shown in Figure 1b. We observed a relatively large signal for $V_{\;yxx}^{2\omega }$, which is about one‐third smaller than that of $V_{\;zxx}^{2\omega }$ and its quadratic dependence on *I* ^ω^. In contrast, $V_{\;xxx}^{2\omega }$ is much smaller and its presence likely results from small projections of all permitted tensor components due to the voltage leads’ misalignment. Furthermore, both $V_{\;yxx}^{2\omega }$ and $V_{\;zxx}^{2\omega }$ are found to be independent of frequency up to 10 kHz (see inset of Figure 1b). They also exhibited similar temperature dependences, with maxima appearing at 315 K, as illustrated in Figure 1c. These results confirm the presence of strong NLHE in CdGeAs_2_ near room temperature. Additionally, the broad peak (valley) observed ≈150 K in the $V_{\;zxx}^{2\omega }$ ($V_{\;yxx}^{2\omega }$) signal may be tentatively attributed to the increment in carrier density, as suggested by the temperature‐dependent carrier density (see Figure S8b, Supporting Information), which contributes to the scattering‐induced NLHE, as discussed below.

We have also estimated the nonlinear Hall angle. θ_*NLAH*_ from the measured transverse and longitudinal second harmonic voltages shown in Figure 1b. θ_*NLAH*_ is defined as $\tan^{-1}\left(V_{\;⊥}^{2w}/V_{\;xxx}^{2w}\right)$. In an ideal Hall device demonstrating an NLHE without mixing of voltage components between the longitudinal and transverse directions, a longitudinal *I* ^ω^ should induce a second‐harmonic Hall voltage response solely along the transverse direction but not along the longitudinal direction, yielding θ_*NLAH*_ = 90°. Figure 1f illustrates the *I* ^ω^‐dependent θ_*NLAH*_ for the CdGeAs_2_ device, showing that the extracted θ_*NLAH*_ remains nearly constant at 89°, indicating that the second harmonic response seen in CdGeAs_2_ is a Hall response. The predominance of the NLHE, along with its frequency independence, linear current‐voltage (*I*–*V*) characteristics of the first harmonic response *V* ^ω^ (see Figure S9, Supporting Information), and nearly linear DC *I*–*V* characteristics measured using the two‐probe method (see Figure S10, Supporting Information) rule out the junction effect and Schottky barriers from the contacts or the thermoelectric effect as the origin of the observed nonlinear transport behavior in the CdGeAs_2_.

In addition to the predominance of the NLHE, we also found that the CdGeAs_2_ device generates a large nonlinear anomalous Hall angle. $\theta_{H}^{A}$. From the symmetrized DC *I*–*V* characteristics of CdGeAs_2_ at 315 K, we observed *V* ^DC^ exhibited quadratic dependence on *I* ^DC^ and reached up to the mV regime, as illustrated in Figure 1d. Using the definition of the anomalous Hall angle $\theta_{H}^{A}=E_{⊥}/E_{\left|\right|}$, where *E*_||_ is obtained by anti‐symmetrizing the DC *I*–*V* characteristics, we plotted the $\tan\theta_{H}^{A}$ as a function of $I_{x}^{DC}$ in Figure 1e. The $\tan\theta_{H}^{A}$ value reached 0.25 and 0.09 at 10 mA, corresponding to $\theta_{H}^{A}$ = 13.9° and 5.4° for *E_zx_* and *E_yx_*, respectively. These Hall angles are significantly larger than previously reported anomalous Hall angles of magnetic conductors at room temperature, such as those observed in MnGa^[^ ^48^ ^]^ and Co_2_MnGa,^[^ ^49^ ^]^ and can be further enhanced by increasing the current.

### 2.2 Electronic Band Structure of CdGeAs_2_

**Figure 2:**
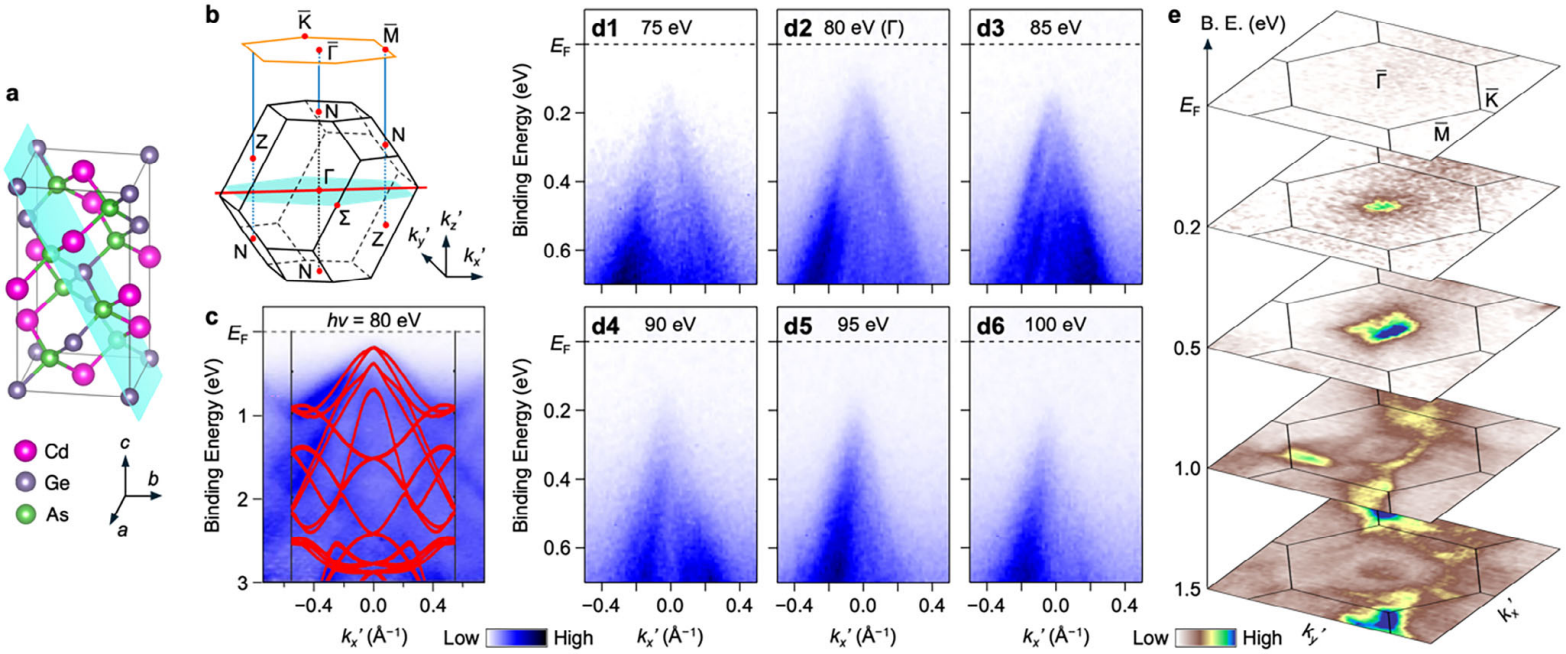
ARPES spectra of CdGeAs_2_. a,b) Schematic crystal structure and Brillouin zone of CdGeAs_2_, respectively. The tiffany blue region indicates a plane parallel to the (101) cleavage surface. c) ARPES intensity plot of the valence‐band region as a function of wave vector along the *k_x_*’ cut and binding energy (*E*_B_), measured at *T* = 30 K with *hν* = 80 eV, compared with the DFT calculation along the same cut (red curves). (**d1‐6**) Photon‐energy dependence of the ARPES intensity near *E*_F_ around the Γ point, signifying a finite band dispersion along out‐of‐plane wave vector, *k_z_*’. e) Equi‐energy contour plots of the ARPES intensity as a function of in‐plane wave vector.

To elucidate the possible contribution of topological nontrivial states to the observed nonlinear transport in CdGeAs_2_ and to clarify its band structure, we performed ARPES measurements using energy‐tunable vacuum‐ultraviolet photons. The cleaved surface corresponds to the (101) crystallographic plane (tiffany blue region in **Figure** **2**a) with the surface normal aligned along the ΓN direction of the Brillouin zone (Figure 2b). For clarity, we define the *k_z_* ′ axis along the ΓN direction and the in‐plane axes *k_x_* ′ and *k_y_* ′ axes as orthogonal to *k_z_* ′ axis, lying within the (101) surface, as depicted in Figure 2b. Figure 2c presents the ARPES intensity along the *k_x_* ′ cut (red line in Figure 2b), acquired at the photon energy *hν* = 80 eV. Several well‐defined dispersive bands are observed in the valence‐band region, indicating successful cleavage and a clean surface. DFT calculation shows overall agreement with the experimental band dispersion, particularly for the topmost Γ‐centered hole‐like valence band, which has its top at a binding energy *E*_B_ of ≈0.2 eV and disperses to ≈1 eV. In addition, other hole‐like dispersions (e.g., topped at *E*_B_ ≈0.4 and 0.8 eV) and electron‐like dispersions (bottomed at ≈1.5 and ≈2.4 eV), including their band widths, are well reproduced. The *Z*_2_ invariant and surface state calculations confirm a topologically trivial ground state (see Note S5, Supporting Information). To investigate the band dispersion along out‐of‐plane wave vector (*k_z_* ′), we performed *hν*‐dependent ARPES measurements near *E*_F_, as shown in Figure 2d. At *hν* = 80 eV, corresponding to a momentum cut through the bulk Γ point, the topmost valence band exhibits a maximum at *E*_B_ of ≈0.15 eV. As *hν* increases from 80 to 100 eV, this band shifts to higher *E*_B_, indicating a finite band dispersion along *k_z_*’ and confirming its bulk origin. Importantly, Figure 2d reveals that the spectral weight above the topmost valence band is significantly suppressed for all the measured photon energies, and no indication of a topological surface state is present. This conclusion is further supported by the constant‐energy contour maps shown in Figure 2e. At *E*_F_, the intensity is negligible, consistent with the absence of metallic states. In contrast, the contour maps at *E*_B_ = 0.2 eV reveal a bright spot at the surface Brillouin‐zone center, corresponding to the bulk valence‐band maximum. These observations confirm the absence of bulk‐band inversion and firmly establish CdGeAs_2_ as a topologically trivial insulator, in stark contrast to previous predictions based on GGA calculations.^[^ ^50^ ^]^ Therefore, our ARPES measurements, supported by DFT calculations, effectively rule out a topologically nontrivial state in CdGeAs_2_. The low electron doping level revealed in transport measurements (Note S3, Supporting Information) is the possible reason why the ARPES experiment did not detect the electron bands.

### 2.3 Scaling Analysis of NLHE

To further elucidate the microscopic origin of the nonlinear transport behavior in CdGeAs_2_, we conducted a comprehensive scaling analysis. Our findings reveal that static disorder scattering together with dynamic scattering predominates in driving the nonlinear response. It is well established that for an intrinsic NLHE originating from a BCD, the second‐order conductivity tensor components *σ_zxx_* and *σ_xzx_* are expected to satisfy the antisymmetric relationship *σ_zxx_* = –*σ_xzx_*.^[^ ^51^ ^]^ However, our experimental data does not conform to this criterion, instead revealing a symmetric relationship *σ_zxx_* = *σ_xzx_* at room temperature, as shown in Figure S11 (Supporting Information).

Furthermore, our symmetry analysis shows that vanishing off‐diagonal components of the BCD tensor preclude a BCD‐driven NLHE, while finite diagonal elements can, in principle, give rise to projected second‐order responses in our experiment setup (see Note S4, Supporting Information). This implies that a contribution from the BCD cannot be ruled out exclusively. However, the dominant NLHE observed at room temperature (Figure 1) cannot be attributed solely to BCD, even though symmetry permits nonzero diagonal tensor elements. This conclusion is strongly reinforced by our scaling analysis below, which establishes that the observed second‐order Hall response in CdGeAs_2_ at room temperature is governed primarily by extrinsic mechanisms.

**Figure 3:**
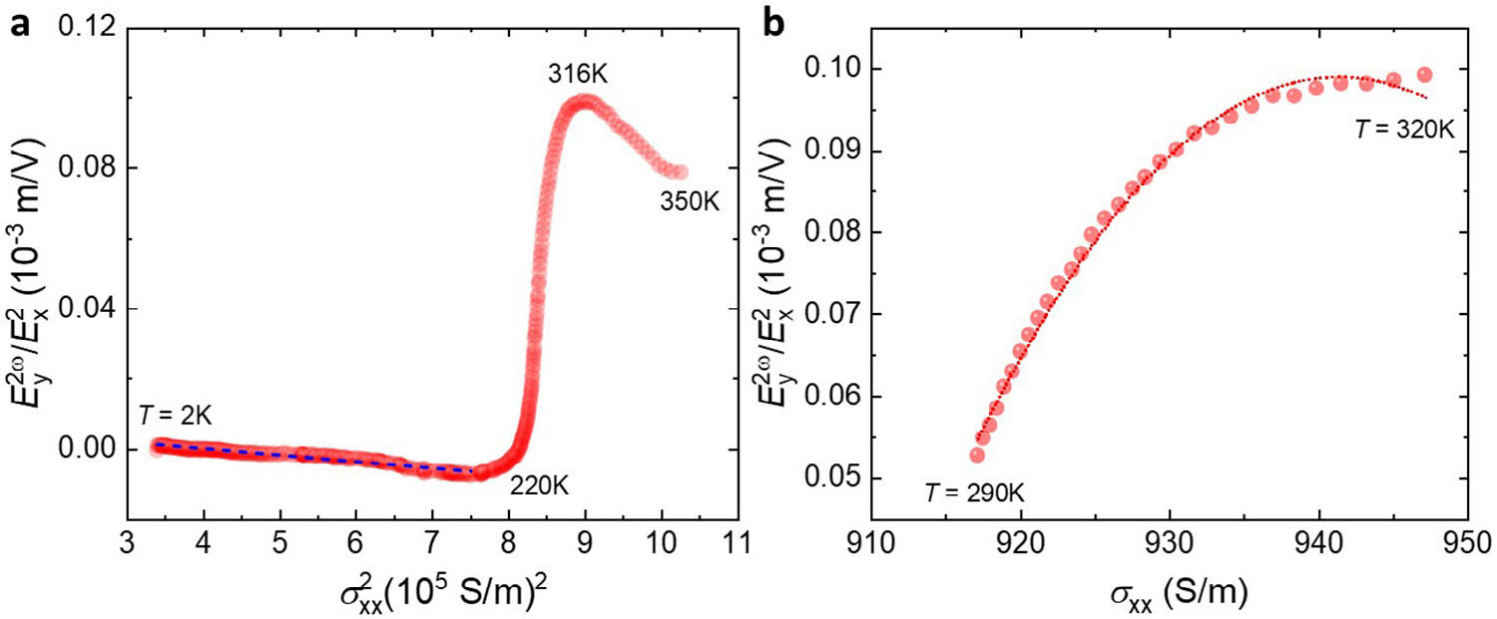
Scaling analysis. NLHE's strength $E_{⊥}^{2\omega }/{\left(E_{\left|\right|}^{\omega }\right)}^{2}$ versus longitudinal bulk conductivity σ_*xx*_ of CdGeAs_2_ at a) 2 to 350 K region and b) 290 to 320 K region. The experimental data points are overlaid with the fit $E_{⊥}^{2\omega }/{\left(E_{\left|\right|}^{\omega }\right)}^{2}=a\sigma_{xx}^{2}+b\sigma_{xx}+c$. Fit coefficients are *a* = −75.5 × 10^−6^ mS/V^2^, *b* = 0.142 × 10^−3^ S/V, *c* = −0.067 m/V. The dashed lines are the fitting.

To confirm this extrinsic origin, we performed a scaling analysis based on the established framework^[^ ^24^, ^30^, ^34^, ^52^ ^]^ under the assumption that static impurity scattering is the solely single source of scattering mechanism dominated at low temperature. This assumption is justified by the weak temperature dependence of the resistivity and saturation of carrier density in this regime (see Figures S4 and S8b, Supporting Information). Since extrinsic contributions scale quadratically with the scattering time *τ*, we analyzed the scaling of the NLHE strength. $E_{⊥}^{2\omega }/{\left(E_{\left|\right|}^{\omega }\right)}^{2}$ as a function of the square of the longitudinal bulk conductivity σ_*xx*_, as shown in **Figure** **3**a. Here, $E_{⊥}^{2\omega }=V_{⊥}^{2\omega }/W$ and $E_{\left|\right|}^{2\omega }=V_{\left|\right|}^{2\omega }/L$ where *W* and *L* denote the transverse and longitudinal voltage probe spacings, respectively. In the temperature range from 2 K to 200 K, the data are approximately fitted by $E_{⊥}^{2\omega }/{\left(E_{\left|\right|}^{\omega }\right)}^{2}=\alpha \sigma_{xx}^{2}+\gamma$, where the intercept *γ* accounts contributions from the BCD^[^ ^1^ ^]^ and/or side‐ jump scattering process^[^ ^4^ ^]^ while the coefficient *α* reflects contribution from skew scattering. The extracted parameters are *α* = ‐1.87 × 10^−11^ m^3^/VS^2^ and *γ* = 7.62 × 10^−6^ m/V. The opposite signs of *α* and *γ* are consistent with previous studies,^[^ ^24^, ^25^, ^31^ ^]^ showing the differing symmetries of skew and side‐jump mechanisms. Importantly, the magnitudes of these contributions are significantly larger than those reported in previously studied room‐temperature nonlinear Hall systems, including Bi_2_Te_3_,^[^ ^25^ ^]^ Te‐thin flakes,^[^ ^25^ ^]^ and MnBi_2_Te_4_ thin films.^[^ ^28^ ^]^ Given that the conductivity of CdGeAs_2_ remains on the order of 10^2^ S/m, the skew scattering term contributes ≈10^−7^ m/V, indicating that side‐jump scattering and/or BCD are the dominant mechanisms governing the nonlinear Hall response at low temperatures.

At elevated temperatures, we observe a sharp increase in $E_{⊥}^{2\omega }/{\left(E_{\left|\right|}^{\omega }\right)}^{2}$, reaching values up to two orders of magnitude larger than those at lower temperatures (see Figure 3a). This enhancement correlates with the increase in carrier density above 150 K (see Figure S8b, Supporting Information), which increases the impact of static impurity scattering. Notably, $E_{⊥}^{2\omega }/{\left(E_{\left|\right|}^{\omega }\right)}^{2}$ exhibits a pronounced peak at 316 K, closely aligned with the peak in carrier density. Simultaneously, dynamic scattering mechanisms, including electron‐phonon and phonon‐phonon interaction, are expected to become increasingly relevant in this temperature regime. Complementary scaling analysis of the transverse nonlinear Hall conductivity $\sigma_{yxx}^{2\omega }$ versus σ_*xx*_ reveals near‐linear dependence below 200 K, consistent with *τ*‐dependent, but strong deviations at higher temperatures (see Note S6, Supporting Information). This deviation suggests that BCD alone, even if present, is insufficient to account for the exceptionally strong NLHE observed near room temperature. Instead, extrinsic mechanisms, dominated by static impurity scattering in conjunction with dynamic scattering processes, govern the remarkably large room‐temperature NLHE observed in CdGeAs_2_.

To account for both static and dynamic scattering contributions, we extended our scaling analysis to the high‐temperature range of 290 K to 320 K, where the second‐order Hall response exhibits a pronounced peak (see Figure 1c), following methodologies established in prior studies.^[^ ^30^, ^31^, ^34^, ^52^ ^]^ Figure 3b illustrates $E_{⊥}^{2\omega }/{\left(E_{\left|\right|}^{\omega }\right)}^{2}$ as a function of σ_*xx*_ and $E_{⊥}^{2\omega }/{\left(E_{\left|\right|}^{\omega }\right)}^{2}$ exhibits a clear temperature dependence, consistent with the earlier report.^[^ ^28^ ^]^ The data are well described by the quadratic fit of the form $E_{⊥}^{2\omega }/{\left(E_{\left|\right|}^{\omega }\right)}^{2}=a\sigma_{xx}^{2}+b\sigma_{xx}+c$, with fitting coefficients *a* = −75.5 × 10^−6^ m^3^/VS^2^, *b* = 0.142 × 10^−3^ m^2^/VS, and *c* = ‐0.067 m/V. These coefficients can be further expressed in terms of different scattering contributions as follows: $a=\left(C_{S}^{sk}\sigma_{xx_{o}}+C_{2}+C_{4}-C_{3}\right)/\sigma_{xx_{o}}^{2}$, $b=\left(C_{3}-2C_{4}\right)/\sigma_{xx_{o}}$, and *c* =  *C*_4_, where $\sigma_{xx_{o}}$ is the residual conductivity, determined at 2 K. The constants *C*_2_, *C*_3_, and *C*_4_ are associated with various scattering mechanisms, such as static impurity scattering $C_{2}=C_{S}^{sj}+C_{SS}^{sk,4}$, mixed scattering $C_{3}=C_{S}^{sj}+C_{ph}^{sj}+C_{Sph}^{sk,4}$, and dynamic electron‐phonon scattering $C_{4}=C_{ph}^{sj}+C_{phph}^{sk,4}$. Here, the subscripts *S* and *ph* refer to static impurities and phonons scattering, while the superscripts *sj*, *sk*, and *sk*,4 correspond to side‐jump, skew, and fourth‐order skew scattering contributions. A semi‐quantitative evaluation yields $C_{S}^{sk}\sigma_{xx_{o}}+C_{2}=0.091$, which notably exceeds both *C*_3_ = 0.050, and *C*_4_ = −0.067. This analysis strengthens the conclusion that static impurity scattering remains the dominant mechanism contributing to the nonlinear Hall response in CdGeAs_2_, even at elevated temperatures where dynamic scattering becomes non‐negligible.

### 2.4 Broadband Electronic Frequency Mixing via NLHE in MHz Range

The nonlinear Hall angle close to 90° and a large anomalous Hall angle observed at room temperature in CdGeAs_2_ highlights its significant potential for advancing technological applications. A key figure of merit in the NLHE system is the NLHE responsivity $R=E_{\;⊥}^{2\omega }/{\left(E_{\;||}^{\omega }\right)}^{2}$. Our measurements reveal that bulk CdGeAs_2_ achieves a record‐high room‐temperature nonlinear Hall responsivity of 2.2 × 10^−3^ m/V, surpassing by ≈60% the highest previously reported value in a textured Pt nanoparticle system^[^ ^32^ ^]^ and exceeding by an order of magnitude the values observed in BaMnSb_2_ and GeTe crystals, which previously exhibited the strong room‐temperature NLHE.^[^ ^8^, ^18^ ^]^

**Figure 4:**
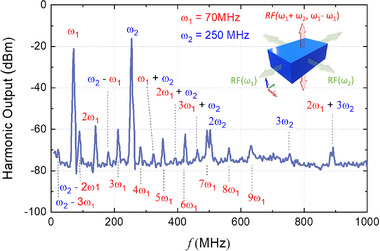
Room temperature broadband electronic frequency mixer features in the MHz range for CdGeAs_2_. Schematic of a diode‐like frequency mixer design using a CdGeAs_2_ device (inset) and its power output mixing spectrum in the MHz range.

Leveraging these exceptional nonlinear transport properties, we demonstrate that CdGeAs_2_ can function as a broadband electronic frequency mixer in the MHz regime. A frequency mixer is capable of combining two input frequencies (ω_1_ and ω_2_) and producing sum and difference frequencies, as well as other higher harmonic frequencies. As shown in the inset of **Figure** **4**, two radio frequency (RF) sources with identical output power but distinct frequencies (VHF: 30–300 MHz) were connected orthogonally to the *x*‐axis and *y*‐axis electrodes of the device. The resulting mixed‐frequency signal was detected along the *z*‐axis. When input frequencies of 70 MHz (*ω*_1_) and 250 MHz (*ω*_2_) were applied, we observed a rich spectrum comprising 19 distinct RF peaks, ranging from 10 to 1000 MHz. These include second‐harmonic generation (2*ω*_1_ and 2*ω*_2_), sum‐frequency generation (*ω*_1_ + *ω*_2_), difference‐frequency generation (*ω*_2_ ‐ *ω*_1_), as well as 16 additional mixed‐frequency components arising from higher‐order nonlinear interactions.

## 3 Summary

Achieving high NLHE responsivity *R* under ambient conditions is crucial for enabling practical technological applications. Our results demonstrate that bulk CdGeAs_2_ fulfills this criterion, exhibiting a record value of nonlinear Hall responsivity of 2.2 × 10^−3^ m/V at room temperature. Through comprehensive investigations, including symmetry and scaling analyses, we identify scattering, particularly from static disorder in conjunction with dynamic scattering, as the dominant mechanism driving the strong room temperature NLHE in CdGeAs_2_. In addition to the strong nonlinear response, we also observe an exceptionally large anomalous Hall angle under direct current excitation at room temperature. Finally, we demonstrate that this strong NLHE facilitates efficient broadband electronic frequency mixing in the MHz range. These findings establish CdGeAs_2_ as a promising platform for nonlinear transport applications and set the foundation for the development of practical devices based on scattering‐induced NLHE in 3D materials.

## 4 Experimental Section

### CdGeAs_2_ Single Crystal Growth

Single crystals of CdGeAs_2_ were synthesized using the Bi‐flux method in the Muffle furnaces with instrument DOI:10.60551/yphx‐tr33. In an argon atmosphere, the source materials of Cd, Ge, As, and Bi‐flux were mixed in the molar ratio of 1:1:2:20. The mixture was placed in an aluminum crucible, evacuated in a sealed quartz tube, and then heated to 1000 °C for 24 h to promote homogeneous melting, and cooled to 750 °C at 2 °C/hour. The Bi‐flux was removed with centrifugation. Bar‐shaped single crystals with a silver luster were obtained using this synthesis procedure. The longest dimension of the crystals is ≈7 mm single crystals (see Figure S1, Supporting Information). Growth and characterization data associated with the samples produced in this study were available via *ScholarSphere*.^[^ ^53^ ^]^

### Electrical Transport and Thermoelectric Measurements

The transport measurements were performed using a commercial Physical Property Measurement System (PPMS, Quantum Design). The standard four‐probe method was used for the transport measurements, in which the current was applied along the $\left[11\bar{1}\right]$ direction on the (112) plane. Field sweeps of Hall resistivity *ρ_xy_* were conducted for both positive and negative fields. The field dependences of *ρ_xy_* were obtained via anti‐symmetrizing the data, i.e., *ρ_xy_* = [*ρ_xy_* (+*µ*_o_ *H*) ‐ *ρ_xy_* (‐*µ*_o_ *H*)]/2.

The thermoelectric measurements were carried out using a home‐built system. A pair of type‐*E* thermocouples was used to measure the sample temperature, with a precision better than 0.01 K.

### Nonlinear Transport Measurements

AC transport measurements were conducted using a Keithley 6221 precision AC/DC current source and Stanford Research SR860 lock‐in amplifiers. DC transport measurements were conducted using a Keithley 2182A nanovoltmeter. All measurements were carried out with PPMS to control the temperatures.

### ARPES Measurements

ARPES measurements with vacuum‐ultraviolet (VUV) photons were conducted at BL7U in UVSOR^[^ ^54^ ^]^ and BL‐28A in PF, KEK,^[^ ^55^ ^]^ using linearly polarized light of 12–40 eV and circularly polarized light of 80–100 eV, respectively. The energy resolution was set at 10–30 meV. Samples were cleaved in situ along the (101) crystal plane, as determined by Laue x‐ray diffraction measurements. Cleanness of the cleaved surface was confirmed by the measurement of core levels in which all the observed peaks were attributed to the Cd, Ge, and As orbitals, and no other core‐level peaks were detected. The sample temperature was kept at *T* = 30 K during measurements. The Fermi level (*E*_F_) of the sample was referenced to that of a gold film electrically contacted with the sample holder. Samples grown by both the Bi flux method were measured and the conventional chemical vapor transport method, confirming the absence of topological surface states in both. Results on the sample grown by the Bi flux method were presented in this manuscript as representative data.

### Density‐Functional Theory and Nonlinear Response Calculations

The band structure of CdGeAs_2_ was computed using the projector augmented wave method as implemented in the VASP package^[^ ^56^, ^57^, ^58^ ^]^ within the GGA scheme.^[^ ^59^ ^]^ The experimental lattice parameters were used. The spin‐orbit coupling was included self‐consistently in the calculations of electronic structures with a Monkhorst‐Pack *k*‐point mesh 15 × 15 × 7. To correct the energy band gaps, calculations with the Heyd–Scuseria–Ernzerhof hybrid functional were also performed.^[^ ^60^ ^]^ A tight‐binding Hamiltonian for chiral‐Te was constructed, where the tight‐binding model matrix elements were calculated by projecting onto the Wannier orbitals, which used the VASP2WANNIER90 interface.^[^ ^61^ ^]^ The Cd‐*s* orbitals, Ge‐*p* orbitals, and As‐*p* orbitals were used to construct the Wannier functions without performing the maximum localization.

## Conflict of Interest

The authors declare no conflict of interest.

## Author Contributions

S.H.L., T.S. and Z.Q.M. conceived the project. S.H.L., Y.W. and K.S. carried out crystal growth. S.H.L. and L.M. performed the transport measurements, and the analysis was performed by S.H.L. and Z.Q.M. The SHG was performed by J.H. and V.G. The ARPES study was performed by T.I., K.N., and T.S. T.Y.L., T.‐R.C., H.L., and L.F. performed the DFT calculations. X.C.P. and Y.P.C. performed the thermoelectric measurements. Z.X. performed the MHz frequency mixing experiment. S.H.L., T.S., and Z.Q.M. wrote the original draft with input from all authors. All authors review and edit the manuscript.

## Supporting information



Supporting Information

## Data Availability

The data that support the findings of this study are openly available in ScholarSphere at, https://doi.org/10.26207/a398‐bq12, reference number 53.
