# Supplementary material for: Disorder Scattering Induced Large Room Temperature Nonlinear Anomalous Hall Effect in a Semiconductor CdGeAs2
Source: Adv Mater. 2025 Nov 23;38(7):e14217. doi: 10.1002/adma.202514217 (PMC12862720; doi:10.1002/adma.202514217)
Supplement: Supplementary file 1 — Supporting Information [file ADMA-38-e14217-s001.pdf]

ADVANCED MATERIALS

Supporting Information

for *Adv. Mater.*, DOI 10.1002/adma.202514217

Disorder Scattering Induced Large Room Temperature Nonlinear Anomalous Hall Effect in a Semiconductor $CdGeAs_2$

Seng Huat Lee, Takumi Iwaya, Kosuke Nakayama, Ting Yong Lim, Lujin Min, Jingyang He, Yu Wang, Venkatraman Gopalan, Zhijian Xie, Xing-Chen Pan, Yong P. Chen, Tay-Rong Chang, Hsin Lin, Liang Fu, Kouji Segawa, Takafumi Sato* and Zhiqiang Mao**

Supplementary information for

Disorder scattering induced large room temperature nonlinear anomalous Hall effect in a semiconductor $CdGeAs_2$

Seng Huat Lee[1,2*], Takumi Iwaya[3], Kosuke Nakayama[3], Ting Yong Lim[4], Lujin Min[2,5], Jingyang He[2,5], Yu Wang[1,2], Venkatraman Gopalan[5], Zhijian Xie[6], Xing-Chen Pan[7], Yong P. Chen[7,8,9], Tay-Rong Chang[4,10,11], Hsin Lin[12], Liang Fu[13], Kouji Segawa[14], Takafumi Sato[3,7*], and Zhiqiang Mao[1,2,5*]

[1]2D Crystal Consortium, Materials Research Institute, The Pennsylvania State University, University Park, Pennsylvania 16802, USA
[2]Department of Physics, The Pennsylvania State University, University Park, Pennsylvania 16802, USA
[3]Department of Physics, Graduate School of Science, Tohoku University, Sendai 980-8578, Japan
[4]Department of Electrical and Department of Physics, National Cheng Kung University, Tainan 70101, Taiwan
[5]Department of Materials Science and Engineering, The Pennsylvania State University, University Park, PA 16802, USA
[6]Department of Electrical and Computer Engineering, North Carolina Agriculture and Technical State University, Greensboro, NC 27411, USA
[7]Advanced Institute for Materials Research (WPI-AIMR), Tohoku University, Sendai 980-8577, Japan
[8]Department of Physics and Astronomy, Elmore Family School of Electrical and Computer Engineering, Birck Nanotechnology Center, Purdue Quantum Science and Engineering Institute, Purdue University, West Lafayette, IN 47906, USA
[9]Institute for Physics and Astronomy and Villum Center for Hybrid Quantum Materials and Devices, Aarhus University, Aarhus-C, 8000 Denmark
[10]Center for Quantum Frontiers of Research and Technology (QFort), Tainan 70101, Taiwan
[11]Physics Division, National Center for Theoretical Sciences, Taipei 10617, Taiwan
[12]Institute of Physics, Academia Sinica, Taipei 115201, Taiwan
[13]Department of Physics, Massachusetts Institute of Technology, Cambridge, MA 02139, USA
[14]Department of Physics, Kyoto Sangyo University, Kyoto 603-8555, Japan

*Email: shl12@psu.edu, t-sato@arpes.phys.tohoku.ac.jp, zim1@psu.edu

This file includes

Note S1. XRD, compositional, and elemental analysis

Note S2. Broken inversion symmetry in $CdGeAs_2$

Note S3. Linear transport properties of $CdGeAs_2$

Note S4. Symmetry analysis for Berry curvature dipole contribution to nonlinear Hall effect

Note S5. DFT calculated band structure and topological state

Note S6. Scaling analysis $\sigma_{yxx}^{2\omega}$ versus σ_{xx}

Note S7. Rectified Hall voltage

Supplementary Figs. S9-S11

Note S1. XRD, compositional, and elemental analysis

Figure S1 shows an XRD pattern measured on the flat surface of a $CdGeAs_2$ single crystal. The sharp (*h h l*) X-ray diffraction peaks indicate the excellent crystallinity of the $CdGeAs_2$ crystal. The composition analysis using EDS reveals that the as-grown single crystal has an atomic ratio of Cd:Ge:As = 0.92:1.07:2.01, which is close to the stoichiometric atomic ratio of $CdGeAs_2$. From the EDS elemental maps in Fig. S2, no cluster of Cd, Ge, and As is observed, suggesting all three elements are uniformly distributed.

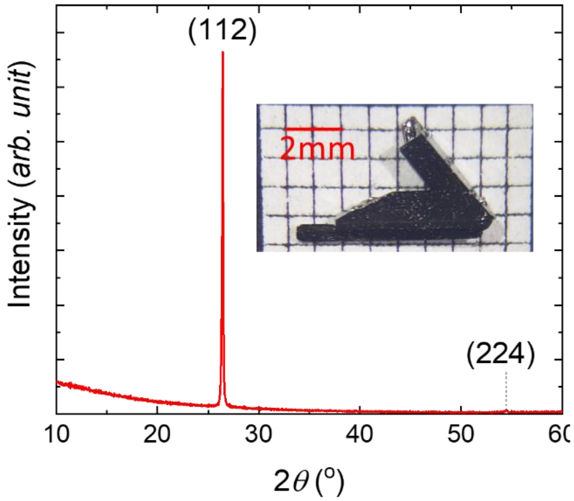

Figure S1. XRD analysis. XRD pattern of $CdGeAs_2$ single crystal probed on the (112) plane. The insets show the optical image of a $CdGeAs_2$ single crystal.

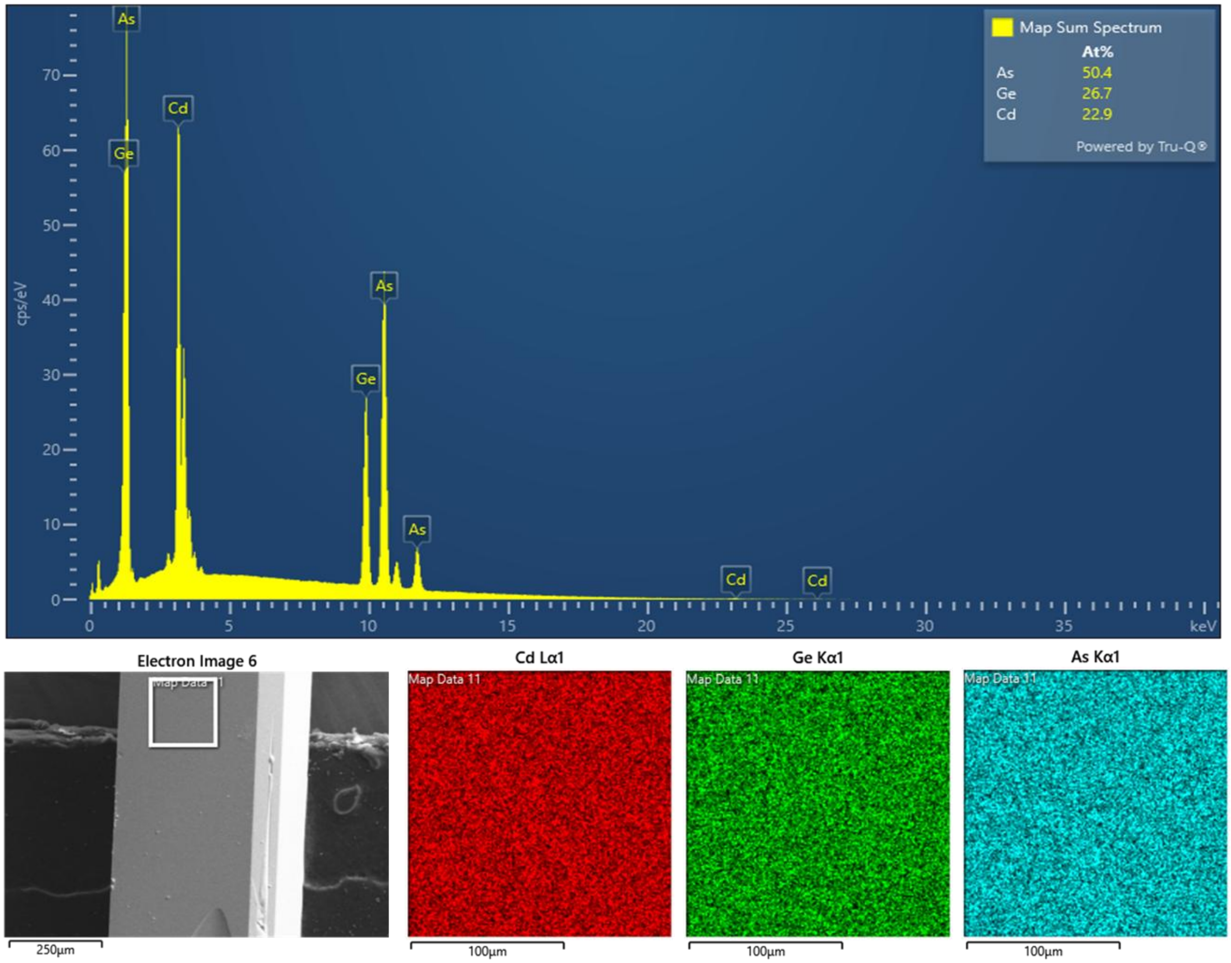

Figure S2. EDS composition analysis. The white box in the SEM micrograph is one of the surveyed areas for composition analysis, and each elemental mapping for Cd, Ge, and As is shown in the bottom panel.

Note S2. Broken inversion symmetry in $CdGeAs_2$

Similar to the prerequisite symmetry for nonlinear optics, the lack of inversion symmetry is also essential for the nonlinear Hall effect (NLHE). To investigate the spatial symmetry of $CdGeAs_2$, we first examined its optical second-order response through second-harmonic generation (SHG) experiments. We conducted SHG measurements on the pristine compound on the (112) plane using femtosecond laser pumping technology at 300 K, as depicted in Fig. S3. The SHG response from $CdGeAs_2$ was measured along the in-plane laboratory axes, with the blue and red lines representing the responses on the *x*- and *y*-axes, respectively, under 800 nm illumination.

Figure S3c shows the significant reflected signal at 400 nm from the $CdGeAs_2$ sample. Notably, the pristine crystal exhibits the symmetry of the point group $\bar{4}2m$ and lacks an inversion center.

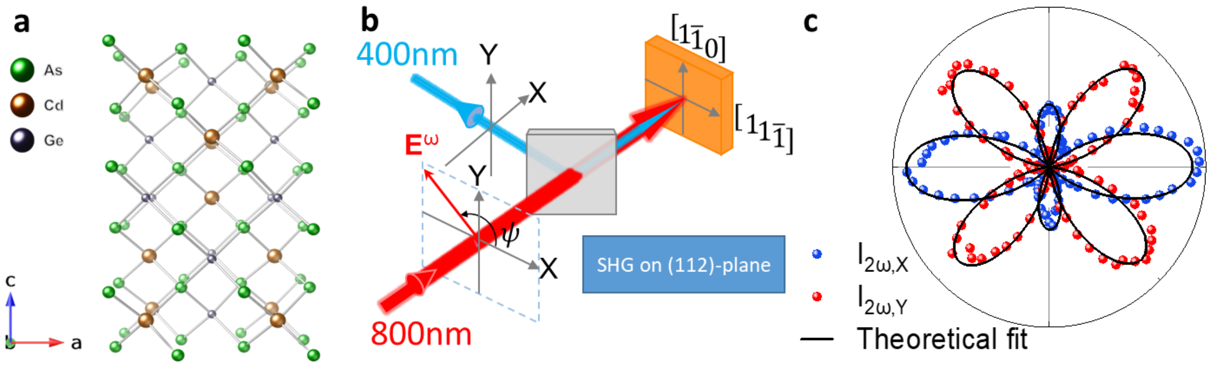

Figure S3. Broken inversion symmetry in $CdGeAs_2$. (**a**) Crystal structure of $CdGeAs_2$. (**b**) The schematic shows the experiment setup for optical SHG measurements on (112) plane of $CdGeAs_2$. **(c)** SHG polar plots of $CdGeAs_2$. Blue and red circles represent the SHG response collected when the analyzer is set with the laboratory x- and y-axis parallel to the $[11\bar{1}]$ and $[1\bar{1}0]$, respectively. The black line is the theoretical fit using an analytical model based on the point group $\bar{4}2m$.

Note S3. Electronic transport properties in $CdGeAs_2$

$CdGeAs_2$ exhibits the highest nonlinear optics (NLO) coefficient among all known mid-infrared NLO materials[1]. However, its practical applications have been limited by significant challenges in achieving low defect-related inhomogeneities, resulting in a predominance of p-type carriers under existing growth methods[2-4]. Previous studies have demonstrated that strong charge compensation through n-type doping can enhance carrier mobility by at least an order of magnitude[2,5].

In this work, we synthesized $CdGeAs_2$ single crystals using a flux growth method, yielding predominantly n-type carrier, as confirmed by Hall effect and Seebeck measurement. The as-

grown crystals exhibit typical carrier densities on the order of 10^{16} cm^{-3} and Hall mobilities exceeding $1000 cm^2/Vs$ (see Fig. S4b), indicating a substantial reduction in defects.

Figure S4a presents the temperature-dependent longitudinal resistivity ρ_{xx} measured with current applied along the $[11\bar{1}]$ direction on the (112) crystallographic plane. Throughout the temperature range of 2 K to 340 K, $CdGeAs_2$ exhibits insulating behavior, with resistivity values in the hundreds of mΩ cm at low temperatures. The Seebeck coefficient further confirms electrons as the dominant charge carriers (see Fig. S4c).

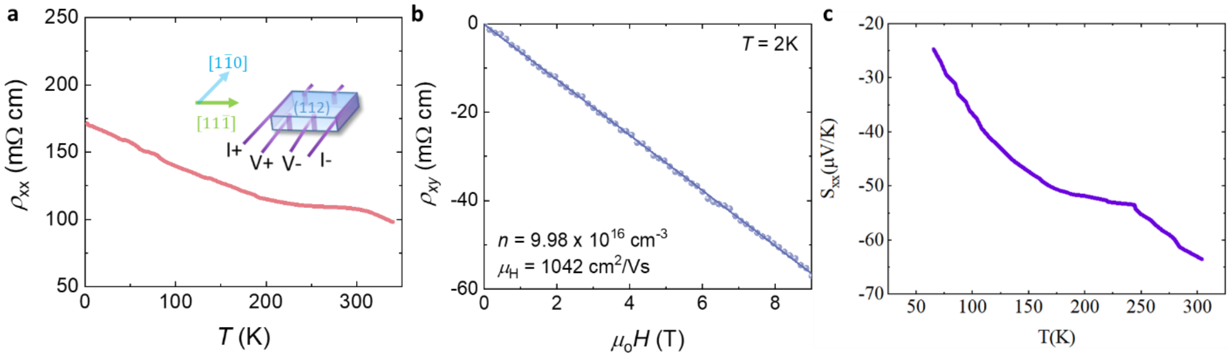

Figure S4. Transport properties of $CdGeAs_2$. (**a**) Temperature dependence of longitudinal resistivity of $CdGeAs_2$. The inset shows the experimental setup for linear transport measurements. The current is applied along the $[11\bar{1}]$ axis. (**b**) Field dependence of Hall resistivity at 2 K. (**c**) Temperature dependence of the Seebeck coefficient from 50 to 300 K, measured with the thermal gradient also applied along the $[11\bar{1}]$ axis.

Note S4. Symmetry analysis for Berry curvature dipole contribution to nonlinear Hall effect

The nonlinear Hall effect is described as $j_a = \sigma_{abc}^{(2)} E_b E_c$, where E is the external electric field, j_a is the nonlinear Hall current density, and $\sigma_{abc}^{(2)}$ is the second-order Hall conductivity.

For the BCD-induced nonlinear Hall effect[6], $\sigma_{abc}^{(2)}$ can be expressed as:

$$\sigma_{abc}^{(2)} = \epsilon_{adc} \frac{e^2\tau}{2(1+\omega\tau)} D_{bd}, \tag{1}$$

Here, ϵ_{adc} denote the Levi-Civita symbol, e is the electron charge, ω is the frequency of the electric field, τ is the relaxation time, and D is the BCD, defined as

$$D_{bd} = \int_k f_k \partial_b \Omega_d \, dk \tag{2}$$

the Ω representing the Berry curvature and f_k is the Fermi-Dirac distribution. We now define the BCD tensor as:

$$D \equiv \begin{pmatrix} D_{aa} & D_{ba} & D_{ca} \\ D_{ab} & D_{bb} & D_{cb} \\ D_{ac} & D_{bc} & D_{cc} \end{pmatrix}$$

In addition to inversion breaking, the BCD tensor is constrained by the crystal point symmetries in the form $D = det(S)SDS^T$, with S being the crystalline symmetry operator[6]. The $CdGeAs_2$ bulk crystal belongs to space group 122, which has C_2 symmetry on the *ab*-plane and *ca*-plane.

Considering the BCD tensor constraints imposed by C_2 symmetry on the *ab*-plane,

$$S = \begin{pmatrix} -1 & 0 & 0 \\ 0 & -1 & 0 \\ 0 & 0 & 1 \end{pmatrix}, \det(S)=1,$$

Thus,

$$D = det(S)SDS^T = \begin{pmatrix} -1 & 0 & 0 \\ 0 & -1 & 0 \\ 0 & 0 & 1 \end{pmatrix} \begin{pmatrix} D_{aa} & D_{ba} & D_{ca} \\ D_{ab} & D_{bb} & D_{cb} \\ D_{ac} & D_{bc} & D_{cc} \end{pmatrix} \begin{pmatrix} -1 & 0 & 0 \\ 0 & -1 & 0 \\ 0 & 0 & 1 \end{pmatrix}$$

$$= \begin{pmatrix} D_{aa} & D_{ba} & -D_{ca} \\ D_{ab} & D_{bb} & -D_{cb} \\ -D_{ac} & -D_{bc} & D_{cc} \end{pmatrix}$$

We obtain

$$\begin{pmatrix} D_{aa} & D_{ba} & D_{ca} \\ D_{ab} & D_{bb} & D_{cb} \\ D_{ac} & D_{bc} & D_{cc} \end{pmatrix} = \begin{pmatrix} D_{aa} & D_{ba} & -D_{ca} \\ D_{ab} & D_{bb} & -D_{cb} \\ -D_{ac} & -D_{bc} & D_{cc} \end{pmatrix}$$

resulting in $D_{ca} = D_{cb} = D_{ac} = D_{bc} = 0$

For C_2 symmetry on the *ca*-plane,

$$S = \begin{pmatrix} -1 & 0 & 0 \\ 0 & 1 & 0 \\ 0 & 0 & -1 \end{pmatrix}, \det(S)=1,$$

which implies

$$\begin{pmatrix} D_{aa} & D_{ba} & D_{ca} \\ D_{ab} & D_{bb} & D_{cb} \\ D_{ac} & D_{bc} & D_{cc} \end{pmatrix} = \begin{pmatrix} D_{aa} & -D_{ba} & D_{ca} \\ -D_{ab} & D_{bb} & -D_{cb} \\ D_{ac} & -D_{bc} & D_{cc} \end{pmatrix}$$

Therefore, $D_{ba} = D_{cb} = D_{ab} = D_{bc} = 0$.

Although the non-diagonal components of the BCD tensor components are zero, the nonzero diagonal components can possibly create a nonlinear response along the *y*-axis in our experiment setup, as discussed below.

To assess the effect of the diagonal components of the BCD tensor, we performed a coordinate transformation from the crystallographic axes (*a*, *b*, *c*) to the experimental frame (*x*, *y*, *z*), where *x* corresponds to $[11\bar{1}]$, *y* corresponds to $[1\bar{1}0]$ and *z* corresponds to [112]. The corresponding relations are

$$x = \frac{1}{\sqrt{3}}(\hat{a} + \hat{b} - \hat{c})$$

$$y = \frac{1}{\sqrt{2}}(\hat{a} - b)$$

$$z = \frac{1}{\sqrt{6}}\left(\hat{a} + \hat{b} + 2\hat{c}\right)$$

Applying this transformation to the BCD tensor and neglecting non-diagonal components in the crystallographic frame, the transformed tensor elements can be expressed as

$$D_{a'b'} = \partial_{a'}\Omega_{b'} \rightarrow \epsilon_{abc} D_{aa} \frac{\partial_a}{\partial_{a'}} \frac{\partial_b}{\partial_{a'}} \frac{\partial_c}{\partial_{c'}} \text{ (with Einstein notation)}$$

Given that the nonlinear Hall conductivity is related to the BCD through[6]

$$\chi_{abc} \propto \epsilon_{adc} D_{bd}$$

with $\chi_{abc} = \frac{j_a}{E_b E_c}$ and $D_{ab} \propto \partial_a \Omega_b$ is the BCD, substitution of the above coordinate relations shows that the second-order conductivity along the z-axis ([112] direction) is zero. This is because the BCD-induced nonlinear response along this direction has both positive and negative contributions and cancel each other under the symmetry constraint $D_{aa} = D_{bb}$. Thus, our observed nonlinear Hall voltage V_{zxx} (Fig.1) should not originate from BCD.

In contrast, the second-order conductivity along the y-axis ($[1\bar{1}0]$ direction) yields

$$\chi_{yxx} \propto D_{xz} \propto -3\sqrt{2}D_{aa} - 3\sqrt{2}D_{bb} + 6\sqrt{2}D_{cc} \neq 0,$$

which means our observed nonlinear Hall voltage V_{yxx} may involve some contributions from diagonal BCD.

This analysis provides a clear picture of how diagonal BCD components contribute to the observed nonlinear Hall response in our device setup. Nevertheless, as shown by our scaling analysis of the nonlinear conductivity with respect to the longitudinal conductivity, the dominant room-temperature NLHE in $CdGeAs_2$ cannot be ascribed solely to the BCD contribution, even though symmetry allows for a nonzero diagonal tensor element.

Note S5. DFT calculated band structure and topological state

The bulk band structure at $k_z = 0$ is shown with orbital decomposition of Cd-*s* orbitals and As-*p* orbitals. The topmost valence band, located at the Γ point, matches well with the ARPES measurements. As illustrated in Fig. S5, the calculated band structure indicates an insulating state with a band gap of 0.5 eV with no evidence of bulk band inversion near the Fermi level. To further probe the topological character, we performed surface state calculations using the surface Green's function technique. In Fig. S6, we show that the semi-infinite surface calculations along the k_x direction reveal the absence of surface states connecting the valence and conduction bands, consistent with a trivial insulating state. Additionally, in the Wilson loop spectrum, the Wannier centers cross any horizontal line is an even number of times (either zero or two), as shown in Fig. S7, thereby confirming the topologically trivial nature of the system.

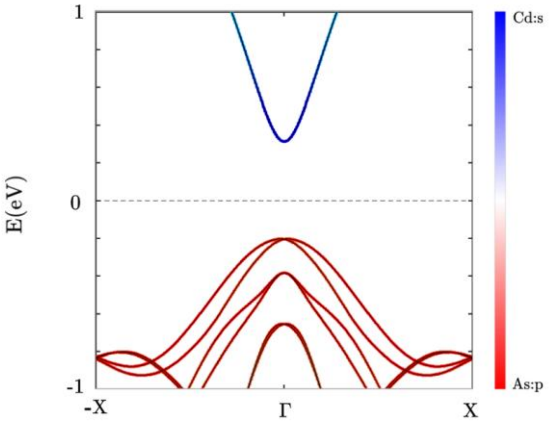

Figure S5. DFT calculated band structure with orbital decomposition of $CdGeAs_2$. DFT calculated bands along high-symmetry directions. Orbitals characters are indicated by the different colors.

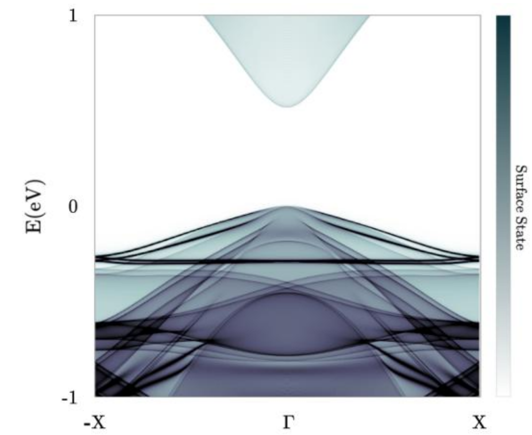

Figure S6. Surface state electronic structure by surface Green's function technique. Semi-infinite surface calculations along the k_x direction reveal no surface states connecting the valence and conduction bands.

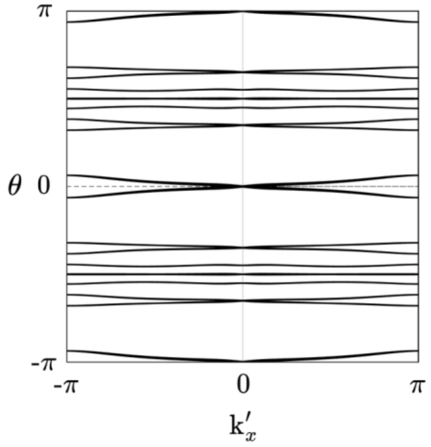

Figure S7. Wilson loop. The Wannier centers cross any horizontal line an even number of times (either zero or two), confirming that the system is a trivial insulator.

Note S6. Scaling analysis $\boldsymbol{\sigma_{yxx}^{2\omega}}$ versus $\boldsymbol{\sigma_{xx}}$

To disentangle the microscopic origins of nonlinear Hall response, scaling analysis has proven to be a powerful tool to distinguish intrinsic mechanisms (BCD and quantum metric) from extrinsic scattering processes (side-jump, skew scattering, and dynamic scattering), as each exhibits a

characteristic dependence on the scattering time τ [7-9]. Specifically, band-geometric contributions such as the quantum metric is independent of τ, BCD-induced response scales linearly with τ ($\propto \sigma_{xx}$), while extrinsic scattering processes is quadratic in τ ($(\sigma_{xx})^2$).

We therefore analyzed the temperature dependence of the transverse nonlinear Hall conductivity $\sigma_{yxx}^{2\omega}$, extracted from the measured nonlinear Hall voltage using

$$\sigma_{yxx}^{2\omega} = j_{yxx}^{2\omega}/(E_{xx}^{\omega})^2 = \frac{V_{yxx}^{2\omega}}{(I_x^{\omega})^2 R_{xx}^3} \frac{l^3}{w^2 t}$$

where $j_{yxx}^{2\omega}$, E_{xx}^{ω}, R_{xx}, l, w, and t are the second-order current density, longitudinal electric field, linear longitudinal resistance, and the length, width, and thickness of the device, respectively. As shown in Fig. S8a, $\sigma_{yxx}^{2\omega}$ scales nearly linearly with σ_{xx} between 2 K and 200 K, consistent with a τ-dependent contribution and indicating that BCD may play a role at low temperatures in addition to extrinsic scattering. However, above 220 K $\sigma_{yxx}^{2\omega}$ strongly deviates from linearity, suggesting that BCD alone cannot account for the high-temperature response. Instead, extrinsic mechanisms, particularly static impurity scattering as well as the dynamic electron-phonon interactions, dominate the NLHE and give rise to the exceptionally large responsivity at room temperature, as discussed below.

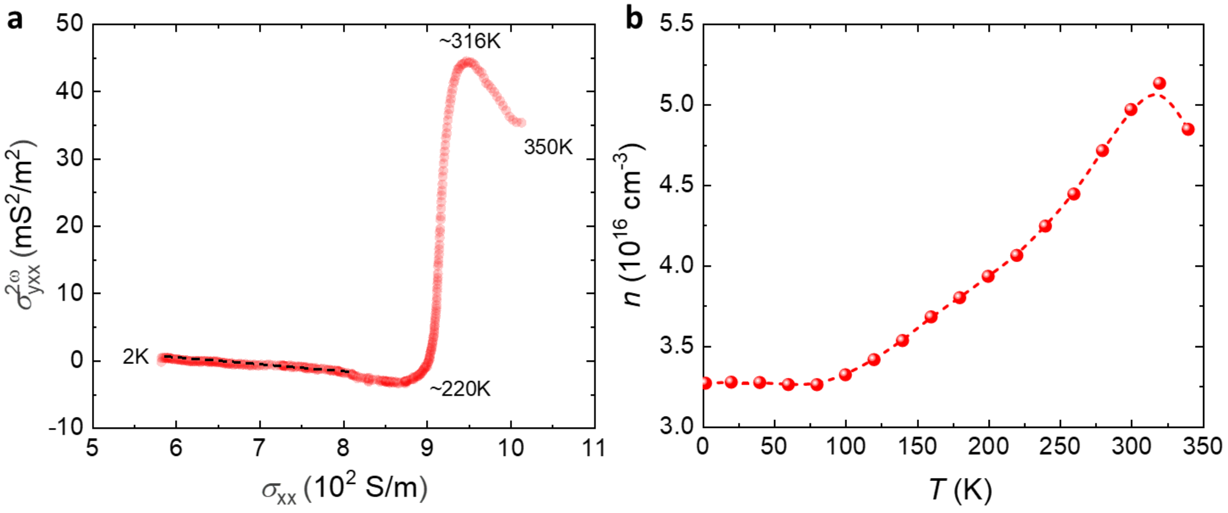

Figure S8. **Scaling analysis.** (**a**) The scaling relationship between the nonlinear Hall conductivity $\sigma_{yxx}^{2\omega}$ and the linear longitudinal conductivity σ_{xx}. The scaling law analysis was carried out within the temperature range of 2 – 350 K. The dashed line is a linear fit of the data from 2 K to 200 K. (**b**) Temperature dependence of Hall carrier density in $CdGeAs_2$. Below 100 K, the carrier density remains nearly temperature-independent, while above 100 K it increases with temperature and exhibits a peak at 320 K.

In Fig. S8a, $\sigma_{yxx}^{2\omega}$ exhibits a pronounced peak near 316 K, which closely correlates with nonmonotonic carrier density evolution. As shown in Fig. S8b, carrier density remains nearly temperature-independent below 100 K but increases as the temperature approaches 320 K before decreasing at higher temperatures, producing a clear, pronounced maximum coincident with the $\sigma_{yxx}^{2\omega}$ peak. Simultaneously, dynamic scattering processes, such as electron-phonon and phonon-phonon interactions, are expected to become increasingly relevant at elevated temperatures. Since we can scale $E_{\perp}^{2\omega}/\left(E_{||}^{\omega}\right)^2$ with respect to $\sigma_{xx}$by considering the static impurity scattering together with the dynamic electron-phonon interactions in the high temperature regime where the second-order response reaches its maximum (Fig. 3b), the extrinsic scattering should be the dominant contribution to the nonlinear Hall response in $CdGeAs_2$ in the high temperature range.

These results clarify that, while BCD contribution is possibly present, the NLHE in $CdGeAs_2$ at room temperature is primarily governed by extrinsic scattering, with static impurities providing the dominant channel and dynamic scattering processes supplying a significant correction. This conclusion supports that the record-high room-temperature nonlinear Hall responsivity originates from extrinsic scattering rather than intrinsic BCD.

It is further worth pointing out that a vanishing BCD does not necessarily imply the absence of an extrinsic scattering mechanism contributing to NLHE. Extrinsic disorder scattering remains a robust mechanism for generating nonlinear Hall currents[7,10-12]. The scattering contribution to the nonlinear Hall effect, arising in the context of chiral Bloch electrons[10,12], has a quantum origin in the intrinsic chirality of electron wavefunctions in time-reversal invariant and inversion-asymmetric materials, which generates asymmetric scattering rates between momentum states $\boldsymbol{k}$ and $\boldsymbol{k'}$ during impurity scattering events. This asymmetry scattering, embodied in the antisymmetric part of the scattering rate, enters the semiclassical Boltzmann transport equation via the collision integral[7], yielding a nonequilibrium distribution function that, when weighted with the electron velocity, produces a nonlinear Hall current.

In contrast to the BCD mechanism, which subjects stringent constraints from point group symmetries and often vanishes in crystals with higher rotational symmetries ($n \geq 3$) or mirror symmetries due to cancellations over the full Brillouin zone[6,7], the scattering effect originates from local asymmetries in wavefunction overlaps at individual scattering sites[10,12], rather than a global integral of the BCD. Under the broken inversion symmetry, the antisymmetric scattering rate remains finite, permitting the chiral Bloch wavefunctions that differ at different $\boldsymbol{k}$[7,11]. Such chirality remains locally even when global symmetries enforce cancellations of net BCD[7,12]. The scattering contribution is therefore allowed in a wide class of noncentrosymmetric crystals, with the sole

requirement being inversion asymmetry to enable finite nonlinear conductivity; higher symmetries (e.g., rotations or mirrors) do not necessarily force it to vanish[7,11,12]. Hence, while both BCD and impurity scattering necessitate inversion asymmetry, the BCD-induced nonlinear Hall effect is constrained by more strict point group symmetry requirements than the scattering contribution.

Note S7. Rectified Hall voltage

The rectified Hall voltage in a nonlinear Hall system refers to a DC Hall voltage that develops along the transverse direction when an AC current is applied longitudinally. This effect originates directly from the nonlinear Hall response: an AC driving current generates two Hall voltage components at zero magnetic field—one DC component and another oscillating component at twice the driving frequency. The DC component is termed the *rectified voltage* because the nonlinear Hall effect effectively converts an oscillating electric field into a steady transverse DC voltage, analogous to the rectification function of semiconductor diodes.

When an AC electric field is applied along the longitudinal *x*-axis, the nonlinear Hall voltage develops along the transverse *y*-axis. The corresponding nonlinear Hall current can be expressed as $j_y(t) = \chi_{yxx} E_x^2(t)$, where χ_{yxx} is the nonlinear Hall conductivity tensor. For an AC excitation $E_x(t) = E_o sin(\omega t)$, this becomes $j_y(t) = \chi_{yxx} E_o^2 sin^2(\omega t) = \frac{\chi_{yxx} E_o^2}{2}[1 - \cos(2\omega t)]$. This decomposition shows that the nonlinear Hall current (and thus the nonlinear Hall voltage) contains two contributions: a constant DC term ($\propto E_o^2/2$) and an oscillating term at twice the drive frequency (2ω). The first term corresponds to the rectified (DC) Hall response. This is the key signature of nonlinear Hall physics, even though the input is purely alternating, the material

produces a steady transverse voltage. Meanwhile, the oscillating term represents the second harmonic Hall response.

Physically, the material functions as a rectifier, it converts an oscillating longitudinal input into a one-way (DC) transverse voltage to produce DC power. This nonlinear rectification effect highlights potential applications in wireless charging and energy harvesting technology.

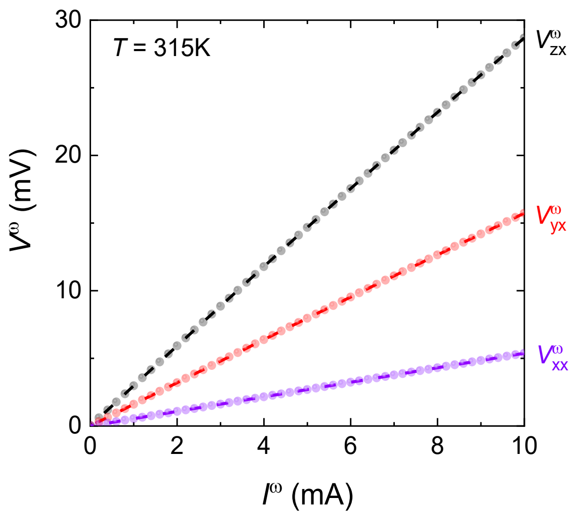

Figure S9. *I-V* characteristic of the first harmonic response in $CdGeAs_2$. AC current-voltage (*I-V*) characteristic measurements on bulk single crystal $CdGeAs_2$ at 315K. The *I-V* characteristics exhibit linear responses across all voltage probes, indicating the formation of reliable ohmic contacts, no contact junction effect, and Schottky barriers are involved in the measurements.

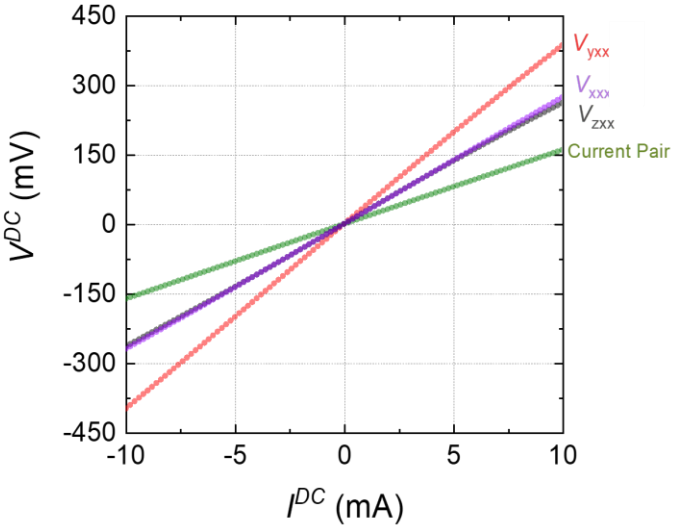

Figure S10. Two-probe *I-V* characteristic of $CdGeAs_2$. Two-probe DC current-voltage (*I-V*) characteristic measurements on the bulk single crystal $CdGeAs_2$ at 300K. All I-V characteristics exhibit nearly linear *I-V* responses, indicating no contact junction effect and Schottky barriers are involved in the measurements.

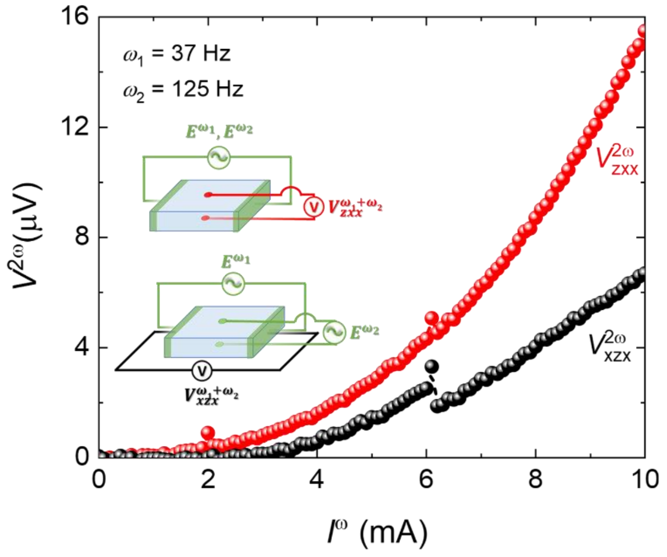

Figure S11. The sum frequency generation measurements. $V^{2\omega}$ as a function of input AC current at 300K. The schematics illustrate the experimental setup for σ_{zxx} (top) and σ_{xzx} (bottom) measurements.

References

1 Zakel, A. *et al.* Temperature and pulse-duration dependence of second-harmonic generation in $CdGeAs_2$. *Appl. Opt.* **41**, 2299-2303 (2002). https://doi.org/10.1364/AO.41.002299

2 Bairamov, B. H., Rud, V. Y. & Rud, Y. V. Properties of Dopants in $ZnGeP_2$, $CdGeAs_2$, $AgGaS_2$ and $AgGaSe_2$. *MRS Bulletin* **23**, 41-44 (1998). https://doi.org/10.1557/S0883769400029080

3 Bairamov, B. K. *et al.* Optoelectronic effects in *p*-$CdGeAs_2$ single crystals and structures based on them. *Physics of the Solid State* **40**, 190-194 (1998). https://doi.org/10.1134/1.1130269

4 Johnson, B. R. *et al.* Synthesis and Characterization of Bulk, Vitreous Cadmium Germanium Arsenide. *Journal of the American Ceramic Society* **92**, 1236-1243 (2009). https://doi.org/https://doi.org/10.1111/j.1551-2916.2009.03001.x

5 Rud', V. Y., Rud', Y. V., Pandey, R. & Ohmer, M. C. Evidence of High Electron Mobility in $CdGeAs_2$ Single Crystals. *MRS Online Proceedings Library* **607**, 439 (2000). https://doi.org/10.1557/PROC-607-439

6 Sodemann, I. & Fu, L. Quantum Nonlinear Hall Effect Induced by Berry Curvature Dipole in Time-Reversal Invariant Materials. *Physical Review Letters* **115**, 216806 (2015). https://doi.org/10.1103/PhysRevLett.115.216806

7 Du, Z. Z., Wang, C. M., Li, S., Lu, H.-Z. & Xie, X. C. Disorder-induced nonlinear Hall effect with time-reversal symmetry. *Nature Communications* **10**, 3047 (2019). https://doi.org/10.1038/s41467-019-10941-3

8 Wang, C., Gao, Y. & Xiao, D. Intrinsic Nonlinear Hall Effect in Antiferromagnetic Tetragonal CuMnAs. *Physical Review Letters* **127**, 277201 (2021). https://doi.org/10.1103/PhysRevLett.127.277201

9 Kaplan, D., Holder, T. & Yan, B. Unification of Nonlinear Anomalous Hall Effect and Nonreciprocal Magnetoresistance in Metals by the Quantum Geometry. *Physical Review Letters* **132**, 026301 (2024). https://doi.org/10.1103/PhysRevLett.132.026301

10 Isobe, H., Xu, S.-Y. & Fu, L. High-frequency rectification via chiral Bloch electrons. *Science Advances* **6**, eaay2497 (2020). https://doi.org/doi:10.1126/sciadv.aay2497

11 Ma, D., Arora, A., Vignale, G. & Song, J. C. W. Anomalous Skew-Scattering Nonlinear Hall Effect and Chiral Photocurrents in *PT*-Symmetric Antiferromagnets. *Physical Review Letters* **131**, 076601 (2023). https://doi.org/10.1103/PhysRevLett.131.076601

12 Atencia, R. B., Xiao, D. & Culcer, D. Disorder in the nonlinear anomalous Hall effect of *PT*-symmetric Dirac fermions. *Physical Review B* **108**, L201115 (2023). https://doi.org/10.1103/PhysRevB.108.L201115
